# CEUS Retrograde Cystography Is Helpful in Percutaneous Drainage of Complex Posttransplant Lymphocele

**DOI:** 10.1155/2012/919215

**Published:** 2012-01-05

**Authors:** Stefano Di Domenico, Valentina Patti, Federico Fazio, Elisabetta Moggia, Iris Fontana, Umberto Valente

**Affiliations:** Department of General Surgery and Organ Transplantation, San Martino University Hospital, Largo Rosanna Benzi 10, 16132 Genoa, Italy

## Abstract

After monolateral dual kidney transplantation, a 69 years old male patient developed symptomatic lymphocele with mild hydroureteronephrosis, impaired renal function, and right inferior limb oedema. A percutaneous ultrasound-guided drainage of the fluid collection was planned, but the complex mutual relations between the collection and the renal hilus did not allow to identify a suitable route for a safe drainage insertion during conventional ultrasound examination. A retrograde cystography using echographic contrast agent was, therefore, performed, and it clarified the position of both ureters and the renal vessels, permitting an harmless ultrasound-guided percutaneous lymphocele drainage. In conclusion contrast-enhanced ultrasound retrograde cystography may be helpful in percutaneous drainage of complex posttransplant lymphocele.

## 1. Introduction

One of the urological complications in kidney transplant recipients is lymphocele which is a fluid collection between the kidney allograft and the bladder that usually develops during the first months after transplantation. Most lymphoceles are asymptomatic and do not require any treatment since spontaneous resolution occurs after a few months. However, larger lymphocele may lead to deterioration of kidney function, hydroureteronephrosis, fever, lymphedema, and deep vein thrombosis of the ipsilateral leg [1].

When complications occur, drainage of the fluid collection is required: percutaneous puncture and drainage of the fluid collection followed by sclerotherapy is considered the first-line modality due to its effectiveness and safety. Percutaneous drainage is usually performed under ultrasound guidance; however, in presence of complex anatomic relationship of the fluid collection with adjacent structures, CT-guidance is often mandatory [2].

In the present paper, we detail our refined technique to treat symptomatic lymphocele in case of complex anatomy: we performed a percutaneous ultrasound-guided drainage of the fluid collection using retrograde cystography with echographic contrast agent to clarify the complex relationships between renal vessels, ureters, and lymphocele after monolateral dual kidney transplantation.

## 2. Procedure

A 69 years old male patient underwent monolateral dual kidney transplantation for chronic renal failure of unknown origin, previously treated with peritoneal dialysis. Kidneys were procured from a 71 years old cadaveric donor and they were considered suitable for dual transplantation based on donor age and histological score [3].

**Figure 1:**
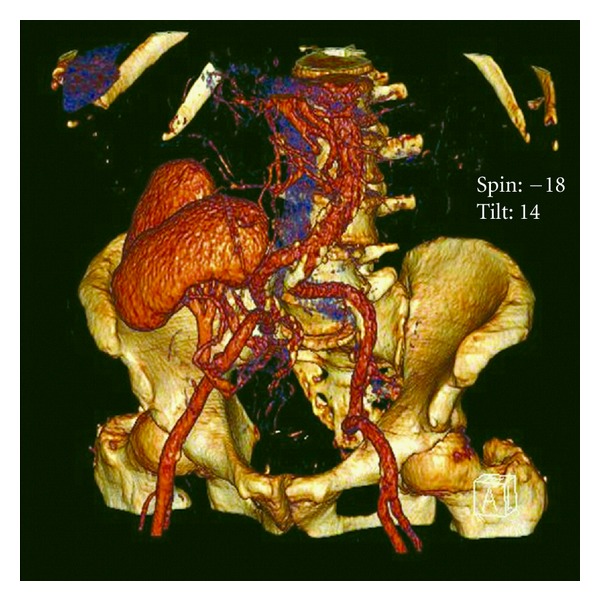
CT 3D reconstruction of the monolateral dual kidney transplantation implanted on the right iliac vessels.

Monolateral dual kidney transplantation was performed with extraperitoneal approach to the right iliac vessels: the left kidney was placed superiorly by anastomosing end-to-side the renal vein and renal artery to the common iliac vessels. After revascularization of the implanted graft, the right kidney was transplanted by anastomosing the renal vessels to the external iliac vessels in end-to-side fashion (Figure 1). Two distinct extravesical ureteroneocystostomies were performed according to the Lich-Gregoir technique with a 6 FR double J stent for each ureter [4, 5].

 Posttransplant period was uneventful, with immediate resumption of diuresis and gradual renal function recovery; standard immunosuppressive treatment was based on FK506, MMF, and prednisone.

After stent removal, patient was discharged in 14th postoperative day (POD) in good clinical conditions with 1.9 mg/dL serum creatinine and 69 mL/min creatinine clearance.

Two months after transplantation, he was referred to our department for impaired renal function with a serum creatinine up to 3.2 mg/dL associated with right inferior limb oedema.

**Figure 2:**
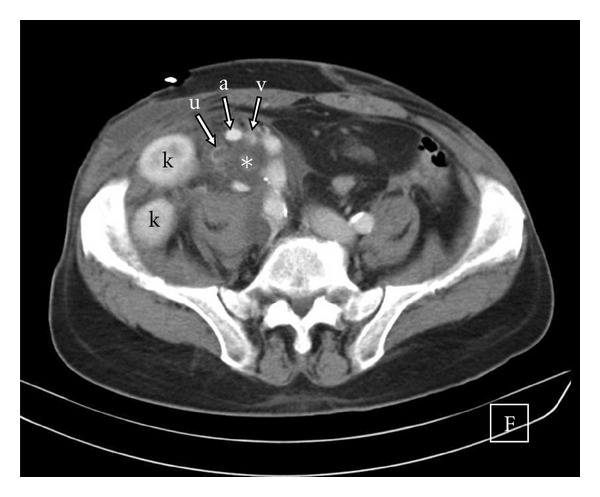
CT scan showing the relationship among the transplanted kidneys (k), the lymphocele (*), the renal artery (a) and vein (v), and the dilated ureter (u).

Urgent ultrasound examination (US) and subsequent CT scan showed a complex fluid collection of 11 cm diameter in the right iliac fossa engulfing both grafts, associated with a mild hydroureteronephrosis of the superior kidney (Figure 2). A Foley's catheter was inserted, and a percutaneous ultrasound-guided drainage of the fluid collection was planned.

 However, the US performed in the operative room did not clearly identify a suitable site for a safe drainage insertion, because of the complex mutual relations among the collection, the renal vessels, and the ureters.

In order to clarify the anatomy, a retrograde cystography using echographic contrast agent was performed using the MyLab 70 US machine with a 1–8 MHz convex probe (Esaote, Genova, Italy): 2.5 mL of SonoVue (Bracco, Milano, Italy) were added to 500 mL of NaCl 0.9% saline solution, and it was instilled in the bladder through a 18 Ch Foley.

**Figure 3:**
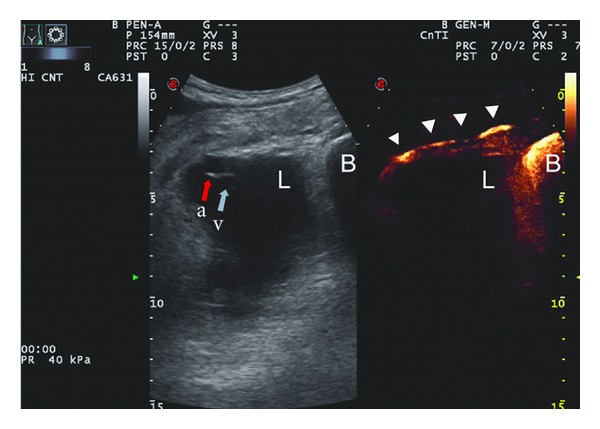
The simultaneous visualization of the same region in conventional B mode and contrast-enhanced mode allowed the identification of ureters. Left side: B mode ultrasound visualization of renal artery (a) vein (v) within the lymphocele (L) and bladder (B). Right side: contrast-enhanced ultrasound visualization of the same region showing the bladder (B) and the ureter (arrows) filled by contrast agent.

Once the bladder was filled by the contrast solution, a minimal vesicoureteral reflux occurred leading to visualization of the terminal ureters by US using the contrast-tuned imaging technique (Figure 3).

Once the position of both ureters was clarified, a 12 Fr drainage catheter was inserted into the collection. Biochemical analysis of the drained fluid confirmed the diagnosis of lymphocele (creatinine of 1.6 g/L, sodium of 137 mEq/L, potassium of 4.9 mEq/L).

**Figure 4:**
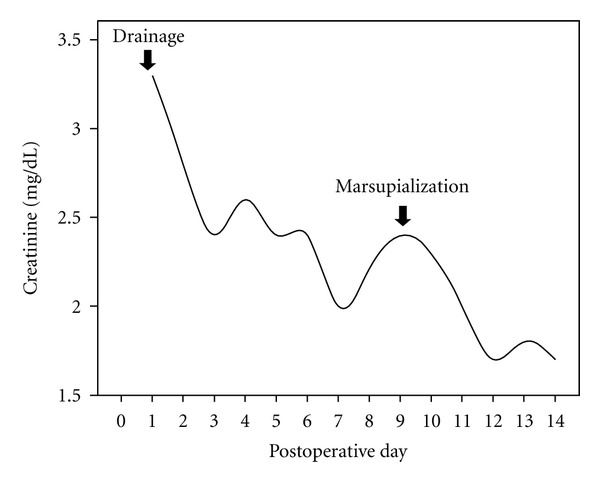
Renal function improved after percutaneous drainage and subsequent marsupialization of lymphocele.

A steady recovery of renal function with decreasing serum creatinine and reduction of the inferior limb edema occurred in the following days (Figure 4). Because of a persistent high volume drainage of 500 mL/day, a surgical marsupialisation was done 8 days after the percutaneous drainage. Subsequent US scan showed resolution of the lymphocele.

 Patient was discharged in 14th POD in good clinical condition with stable renal function.

## 3. Discussion

Lymphocele after kidney transplantation is a common complication ranging from 0.6% to 18%: most of them are asymptomatic and do not require any treatment. Larger fluid collections (more than 3 cm diameter) may lead to hydronephrosis and lessening renal function, they may also cause pain, swell of abdominal wall, ipsilateral leg and scrotum/labium edema, bladder obstruction, and deep vein thrombosis [6].

In our patient a percutaneous drainage of the lymphocele was planned because of the presence of impaired renal function associated with mild ureteronephrosis and inferior limb edema; however, the complex anatomy did not allow a correct identification of both ureters, and therefore it posed a high risk of iatrogenic injuries.

Contrast-enhanced ultrasound (CEUS) voiding cystourethrography is a safe procedure, and it is considered the gold standard to evaluate vesicoureteral reflux in children [7].

We used the same technique to fill the bladder with echographic contrast agent: the presence of a minimal vesicoureteral reflux enabled us to correctly identify the terminal ureters using the flash-echo imaging technique and to perform a safe ultrasound-guided percutaneous drainage. To our knowledge this is the first case reported in the literature of a percutaneous drainage procedure guided by CEUS retrograde cystography.

In conclusion CEUS retrograde cystography may be helpful in percutaneous drainage of complex posttransplant lymphocele.
